# Comparison of the Metabolomics of Different *Dendrobium* Species by UPLC-QTOF-MS

**DOI:** 10.3390/ijms242417148

**Published:** 2023-12-05

**Authors:** Tingting Zhang, Xinxin Yang, Fengzhong Wang, Pengfei Liu, Mengzhou Xie, Cong Lu, Jiameng Liu, Jing Sun, Bei Fan

**Affiliations:** 1 Key Laboratory of Agro-Products Processing, Ministry of Agriculture and Rural Affairs, Institute of Food Science and Technology, Chinese Academy of Agricultural Sciences, Beijing 100193, China; zhang680690@163.com (T.Z.); 13231667362@163.com (X.Y.); wangfengzhong@caas.cn (F.W.); 18774886754@163.com (P.L.); lucong@caas.cn (C.L.); liujiameng@caas.cn (J.L.); 2 Hunan Engineering Technology Research Center for Medicinal and Functional Food, Hunan University of Chinese Medicine, Changsha 410208, China; xiemengzhou@hnucm.edu.cn

**Keywords:** different species, *Dendrobium*, UPLC-QTOF-MS/MS, metabolomics, terpene and flavonoid components, cysteine and methionine metabolism

## Abstract

*Dendrobium* Sw. (family Orchidaceae) is a renowned edible and medicinal plant in China. Although widely cultivated and used, less research has been conducted on differential *Dendrobium* species. In this study, stems from seven distinct *Dendrobium* species were subjected to UPLC-QTOF-MS/MS analysis. A total of 242 metabolites were annotated, and multivariate statistical analysis was employed to explore the variance in the extracted metabolites across the various groups. The analysis demonstrated that *D. nobile* displays conspicuous differences from other species of *Dendrobium*. Specifically, *D. nobile* stands out from the remaining six taxa of *Dendrobium* based on 170 distinct metabolites, mainly terpene and flavonoid components, associated with cysteine and methionine metabolism, flavonoid biosynthesis, and galactose metabolism. It is believed that the variations between *D. nobile* and other *Dendrobium* species are mainly attributed to three metabolite synthesis pathways. By comparing the chemical composition of seven species of *Dendrobium*, this study identified the qualitative components of each species. *D. nobile* was found to differ significantly from other species, with higher levels of terpenoids, flavonoids, and other compounds that are for the cardiovascular field. By comparing the chemical composition of seven species of *Dendrobium*, these qualitative components have relevance for establishing quality standards for *Dendrobium*.

## 1. Introduction

*Dendrobium* Sw. is a genus of valuable medicinal herbs in the family Orchidaceae [1], which has been used in the preparation of herbal medicines in China for more than 2000 years. It is considered a valuable folk medicine with a variety of medicinal activities in traditional Chinese medicine theory, including promoting gastric fluid, nourishing yin, and clearing heat [2,3,4,5]. *Dendrobium* species are rich in polysaccharides, alkaloids, phenanthrenes, bibenzyl, and other bioactive substances with important pharmacological effects [6,7]. Modern pharmacological studies have demonstrated that the *Dendrobium* genus has various functions in the human body, such as immune regulatory, antioxidant, antitumor, antiplatelet, vasodilatory, and hypoglycemic activity [8,9,10].

*Dendrobium* plants are associated with different species in plant taxonomy; therefore, there are large variations in practice. *Dendrobium* contains 74 species and 2 varieties [11] in China, of which many species can be used as medicines. Studies have shown that *Dendrobium. nobile* Lindl. (DN) alkaloids and terpenoids are representative components of these plants, with the functions of nerve protection, angiogenesis promotion, and anti-aging effects [12,13,14]. The *D. officinale* polysaccharides within the plants are highly concentrated, providing significant benefits for hypoglycemia reduction, skin moisturization, and antioxidant effects [15,16,17,18]. The concentration of *D. fimbriatum* bibenzyl components is high, with superior effects in stomach mucous membrane protection and other areas [19]. However, there are numerous types of *Dendrobiums* found in China. Currently, there has been no systematic analysis of the variance in chemical composition across the various *Dendrobium* varieties, resulting in market confusion and challenging identification of medicinal and edible *Dendrobium*. 

*D. officinale* is a highly sought-after commodity in the market due to its usage as a food and medicine. However, the cultivation of *D. officinale* requires significant investment, resulting in a prolonged production cycle. This has led to the influx of numerous *Dendrobium* plants with similar morphology in the domestic medicinal materials market, which are being sold under false pretenses. As a result, there have been attempts to replace *D. officinale* with other types of *Dendrobiums*. Nonetheless, the metabolites present in food and natural herbs can vary depending on the varietal forms used, and this can greatly impact their quality and efficacy. Therefore, it is imperative to tackle uncertainties concerning the divergence in metabolites and their bioactivities across *Dendrobium* cultivars.

In the last few decades, metabolomic analysis based on modern separation science has been developed to investigate biosamples with complex chemical information [20,21]. Metabolomic analyses have exhibited considerable potential for the analysis of food and medicine, as well as for characterizing metabolic variation in terms of intra- and inter-species differences among plants when integrated with multivariate statistical analysis [22,23,24]. In this study, to investigate the compositional differences between seven different varieties of *Dendrobium*, UPLC-QTOF-MS/MS was used to analyze the chemical constituents of the seven varieties *D. denneanum* Kerr. (DD), *D. chrysotoxum* Lindl. (DC), DN, *D. fimbriatum* Hook. (DF), *D. thyrsiflorum* Rchb. f. (DT), *D. officinale* Kimura. et Migo. (DO), and *D. devonianum* Paxton. (DV), and the differences in metabolites among the seven species were determined by a series of chemometric analyses. 

## 2. Results

### 2.1. Annotation of Chemical Constituents

**Figure 1:**
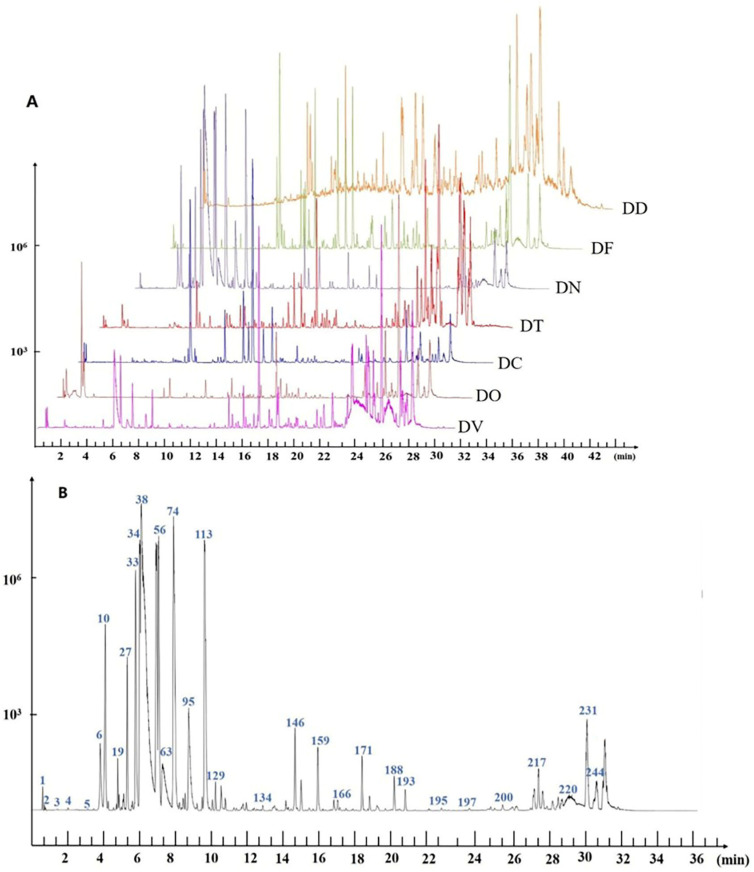
Representative total ion current (TIC) chromatograms of different species of DD (*Dendrobium denneanum* Kerr.), DC (*D. chrysotoxum* Lindl.), DN (*D. nobile* Lindl.) DF (*D. fimbriatum* Hook.), DT (*D. thyrsiflorum* Rchb. f.), DO (*D. officinale* Kimura. et Migo.), DV (*D. devonianum* Paxton.) (**A**) and of DN alone (**B**) under positive ionization mode.

The total ion chromatograms (TIC) representative of the seven species of *Dendrobium* in negative and positive ion modes displayed distinctive components in their ion detection patterns. It was observed that the negative ion mode had a higher sensitivity towards phenolic acids, terpene acids, and flavonoids, attributable to the presence of acid groups. However, the degree of response for a chromatographic peak is higher in positive ion mode than in negative ion mode. Therefore, in this study, we used the positive ion mode for analysis. When combined with the positive ion mode, as demonstrated in Figure 1A, the representative Base peak ion (BPI) chromatogram in the low- Collision energy (CE) mode reveals the entire profile of chemical constituents in *Dendrobium*. The majority of the compounds, including several minor compounds, were detected well, resulting in the probable identification of a total of 242 compounds. The TIC corresponding to the DN is utilized as an example in Figure 1B to analyze the compounds corresponding to the various peaks. The TICs of the other varieties are shown in Figure S1.

#### 2.1.1. Phenolic Acids and Terpene Acids

**Figure 2:**
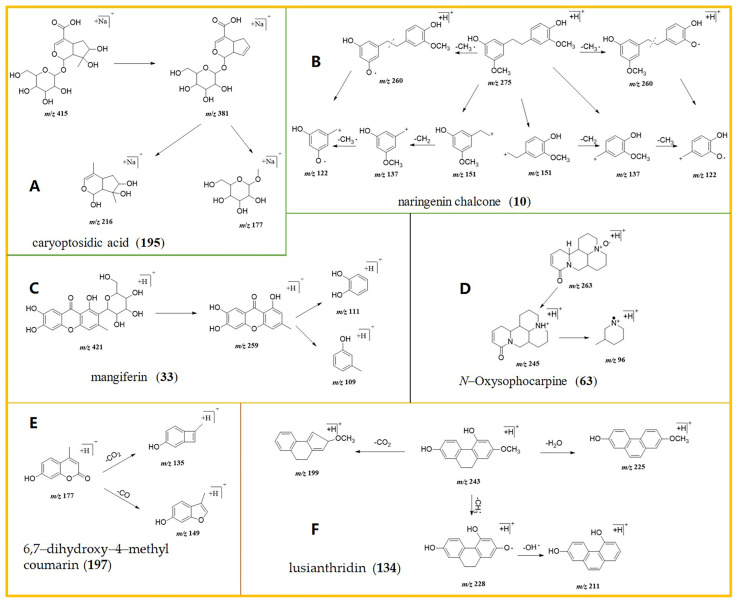
Proposed schematic of the mass fragmentation pathways of (**A**) (compound **195**, caryoptosidic acid); (**B**) (compound **10**, naringenin chalcone); (**C**) (compound **33**, mangiferin); (**D**) (compound **63**, *N*-Oxysophocarpine); (**E**) (compound **197**, 6,7-dihydroxy-4-methyl coumarin); and (**F**) (compound **134**, lusianthridin).

A total of 30 phenolic and terpene acids were annotated. The sodium adduct ion and protonated molecular ion of compound **193** were observed at *m*/*z* 307.0593 ([M + Na]^+^) and 285.0780 ([M + H]^+^), respectively, agreeing well with the molecular formula of C_18_H_12_O_3_. By examining the literature on the retention time, UV spectra, and standard plots, it was concluded that the annotated compound is phenethyl caffeate [25]. For compound **129**, the protonated ion and sodium adduct molecular ion of levistilide A [26] were observed at *m*/*z* 381.2082 ([M + H]^+^) and *m*/*z* 191.1070 ([M + H]^+^), respectively, corresponding to the molecular formula of C_24_H_28_O_4_. Another cyclic enol ether glycoside component, **195**, with *m*/*z* 381.1278 ([M + Na]^+^), *m*/*z* 216.0057 ([M + Na]^+^) and *m*/*z* 177.0102 ([M + Na]^+^) has been reported in the literature as caryoptosidic acid [27]; the cleavage is shown in Figure 2A.

#### 2.1.2. Characterization of Flavonoids

A total of 30 flavonoid constituents were detected in seven extracts of *Dendrobium*, primarily comprising isoflavones, chalcones, and flavonoid glycosides. For compound **10**, the prominent product ions were yielded at *m*/*z* 151.0043 (element composition: C_9_H_10_O_2_), *m*/*z* 122.0517 (element composition: C_7_H_5_O_2_), and *m*/*z* 137.0443 (element composition: C_8_H_8_O_2_); combined with the literature and the corresponding cleavage pattern of the standard, this was presumed to be naringenin chalcone [28]. The fragmentation and cleavage modes are shown in Figure 2B. The product at *m*/*z* 253.1951, which was generated from the quasi-molecular ion *m*/*z* 271.2056 ([M + H]^+^) of compound **159**, was calculated as C_15_H_10_O_5_, corresponding to the cation of galangin [29].

#### 2.1.3. Saccharides and Glycosides

Eight glycosides were annotated, including one terpene glycoside, three glucosides, one oligosaccharide, and three saponins. In the case of compound **31**, the sodium adduct ion and protonated molecular ion of trichothecene isoflavone-7-*O-β*-D-glucoside [30] were observed at *m*/*z* 285.0750 (element composition: C_16_H_12_O_5_) and *m*/*z* 163.1021 (element composition: C_6_H_10_O_5_), respectively, agreeing well with the molecular formula of C_22_H_12_O_10_. Similarly, the sodium adducts ion and protonated molecular ion of mangiferin (compound **33**) [31] were observed at *m*/*z* 259.0817 (element composition: C_14_H_10_O_5_) and *m*/*z* 111.1107 (element composition: C_6_H_6_O_2_), respectively, agreeing well with the molecular formula of C_20_H_20_O_10_ (Figure 2C). 

#### 2.1.4. Alkaloid Characterization

Alkaloids are naturally occurring compounds containing nitrogen with a complex ring structure. The hydrogen atoms in alkaloids often possess lone pairs of electrons, facilitating the possibility to easily lose an electron or gain a proton. Taking compound **8** as a representative to describe the structural characterization process, the positive quasi-molecular ion ([M + H]^+^) was generated at *m*/*z* 322.1074, suggesting a molecular formula of C_19_H_16_NO_4_, and the fragment ions were observed at *m*/*z* 307.0855 and 282.2075 [32]. Compound **146** is presumed to be berberrubine, similar to compound **95**, generated at *m*/*z* 358.1052 ([M + H]^+^), suggesting a molecular formula of C_20_H_18_NO_4_; the fragment ions were observed at *m*/*z* 343.0813, 271.2056, and 253.1951, presumed to be an epiberberine [33]. Compound **63**, presumed to be an *N*-Oxysophocarpine [34], was observed at *m*/*z* 263.1765 ([M + H]^+^), *m*/*z* 96.2376 ([M + H]^+^) and *m*/*z* 245.1665 (element composition: C_15_H_20_N_2_O), agreeing with the molecular formula of C_15_H_22_N_2_O_2_. The fragmentation and cleavage modes are shown in Figure 2D.

#### 2.1.5. Coumarin-like Compounds

Fourteen coumarin-like compounds were hypothesized to be present in the seven *Dendrobium* species, primarily in DN and DF. These compounds generated a robust positive ion mode response, with strong molecular ion peaks predominating in the mass spectra. The spectra frequently exhibit consecutive fragment ion peaks caused by the loss of –CO, –OH or H_2_O, –CH_3_ or –OCH_3_, and alkyl chains. The –CO on the ring is more easily lost than that in the ester bond. Technical term abbreviations are explained in [35]. For example, the sodium adduct ion and protonated molecular ion of compound **38** were observed at *m*/*z* 178.0271 ([M + H]^+^), *m*/*z* 158.0733 ([M + H]^+^), *m*/*z* 150.0324 ([M + H]^+^), and *m*/*z* 133.0286 ([M + H]^+^), in the order of dropping one molecule of methyl and dropping one molecule of –CO and –OH [36]. The sodium adduct ion and protonated molecular ion of compound **197**, presumed to be 6,7-dihydroxy-4-methyl coumarin [37], were observed at *m*/*z* 177.0550 ([M + H]^+^), *m*/*z* 135.0288 ([M + H]^+^), and *m*/*z* 149.0390 ([M + H]^+^), inferred to be 7-hydroxy-4-methyl coumarin (Figure 2E).

#### 2.1.6. Phenanthrene and Quinone Compounds

Compound **56**, with a molecular formula C_15_H_12_O_4_, yielded fragment ions *m*/*z* 258.0156 ([M + H]^+^) and *m*/*z* 241.0892 ([M + H]^+^) and was presumed to be fimbrial-B [38]. Compound **134** was observed at *m*/*z* 211.1020 (element composition: C_13_H_11_O_2_)) and *m*/*z* 228.0446 (element composition: C_14_H_13_O_3_), agreeing well with the molecular formula of C_15_H_14_O_3_ (Figure 2F), and was predicted to be lusianthridin [39]. 

#### 2.1.7. Other Compounds 

The sodium adduct ion and protonated molecular ion of Octyl gallate (compound **1**) were observed at *m*/*z* 305.1372 ([M + H]^+^), agreeing well with the molecular formula of C_15_H_22_O_5_. Compound **113** was observed at *m*/*z* 200.0714 ([M + H]^+^), agreeing well with the molecular formula of C_12_H_9_NO_2_, presumed to be dictamnine. Compound **88** is salsolinol, a heterocyclic compound with *m*/*z* 80.1028 ([M + H]^+^). Compound **171** (pterostilbene (116) +OH+SO_3_) is a heterocyclic benzodiazepine compound of *m*/*z* 256.0992 ([M + H]^+^) and *m*/*z* 257.0111 ([M + H]^+^), which is in agreement with the reported literature [40]. 

### 2.2. Statistical Analysis

#### 2.2.1. Principal Component Analysis (PCA)

**Figure 3:**
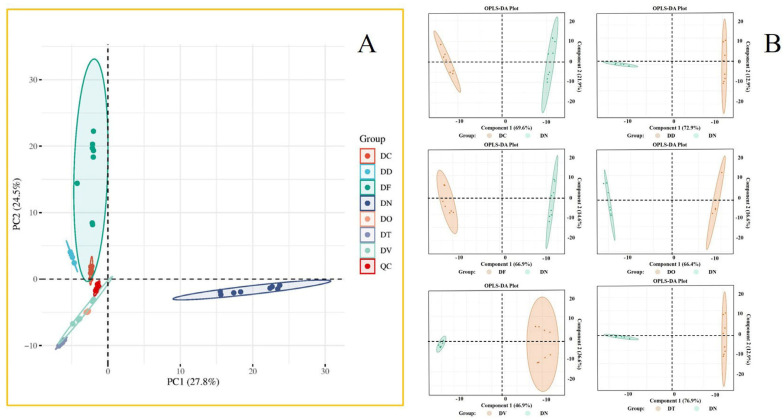
Principal component analysis of different species of DD (*Dendrobium denneanum* Kerr.), DC (*D. chrysotoxum* Lindl.), DN (*D. nobile* Lindl.), DF (*D. fimbriatum* Hook.), DT (*D. thyrsiflorum* Rchb. f.), DO (*D. officinale* Kimura. et Migo.), DV (*D. devonianum* Paxton.) (**A**); a comparison of verified DN and other species of *Dendrobium* (**B**).

The PCA score plots can illustrate discrepancies amongst the diverse *Dendrobium* specimens. As depicted in Figure 3A, the seven samples were categorized into several groups according to the two-dimensional plot of principal components 1 and 2. A particular DF sample manifested outside the 95% confidence interval and could be regarded as an outlier, possibly resulting from raw material inconsistencies or preservation protocols. The PCA score plot illustrates that the QC samples were clustered in the center and tightly distributed, implying that the UPLC-Q-TOF-MS method is reproducible and stable. Thus, the acquired data can be deemed trustworthy and employed for *Dendrobium* metabolomics analysis. Moreover, the samples belonging to the same species were grouped, while the samples from different species were widely scattered and distinguishable in terms of clustering, revealing dissimilarities in the metabolites of various groups of samples concerning their species, amount, and concentration. The PCA score plot is effective in illustrating outliers, groups, and other studied sample types.

#### 2.2.2. DN vs. Other Species of *Dendrobium*—OPLS–DA Analyses

OPLS-DA is a commonly used technique for analyzing metabolomic data and is often applied to differential metabolite screening to enhance intergroup distinctions. In observing the intragroup differences among samples of different origins, the PCA score plot revealed that DN differed more significantly from other *Dendrobium* species. On this basis, further OPLS-DA analysis was performed, and the differences between DN and other varieties of *Dendrobium* were more obvious. Their OPLS-DA scores are plotted in Figure 3B. The OPLS-DA validation diagram is shown in Figure S2.

#### 2.2.3. Differential Screening of DN with Other Species of *Dendrobium*

**Figure 4:**
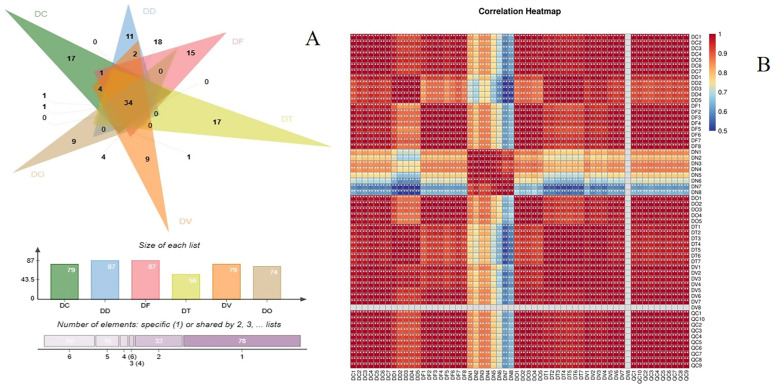
A Venn diagram of variance components (**A**); a heat map of the differential compositions (**B**).

The screening criterion employed was Variable importance in projection (VIP) ≥ 1. The potential marker for *Dendrobium* samples of varying species is heightened with an increasing value of VIP. Components with higher VIP values exert greater influence on the quality difference between *Dendrobium* species and, similarly, on the difference in content between groups. Based on VIP ≥ 1 and *p* ≥ 0.05, a total of 170 distinct components were annotated between DN and other *Dendrobium* species. As shown in Figure 4 and Figure S3, DN is distinguishable from other species of *Dendrobium*, with 79 statistically significant differential components compared to DC, 87 compared to DD, 87 compared to DF, 79 compared to DT, 79 compared to DV, and 74 compared to DO. A total of 34 components were shared in DN from the Venn diagram and heat map, unlike in any other variety of *Dendrobium*. Further analysis (Table 1) revealed that the differential components were primarily terpenes, followed by alkaloids and flavonoids.

#### 2.2.4. Differential Component Pathway Screening

**Figure 5:**
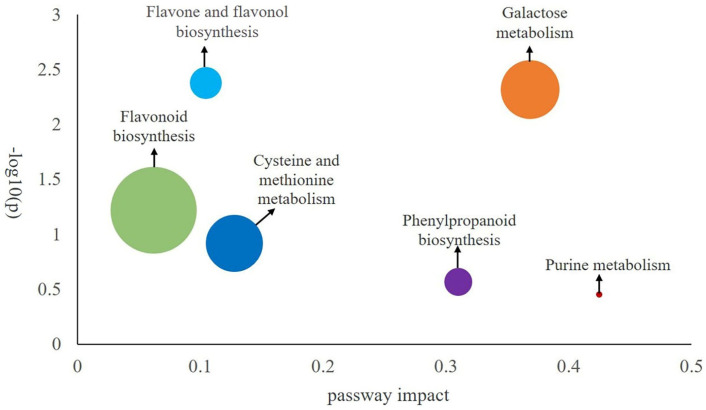
The outcomes of the metabolic pathway analysis. The main differentiating metabolites were annotated and analyzed about metabolic pathways. Each bubble on the bubble diagram represents a metabolic pathway. Technical term abbreviations are explained upon their first use. The horizontal axis and the size of the bubble indicate the size of the pathway influencing factor in the topology analysis. The greater the scale, the bigger the influencing factor. The vertical position of the bubble shows the *p*-value of the enrichment analysis.

**Figure 6:**
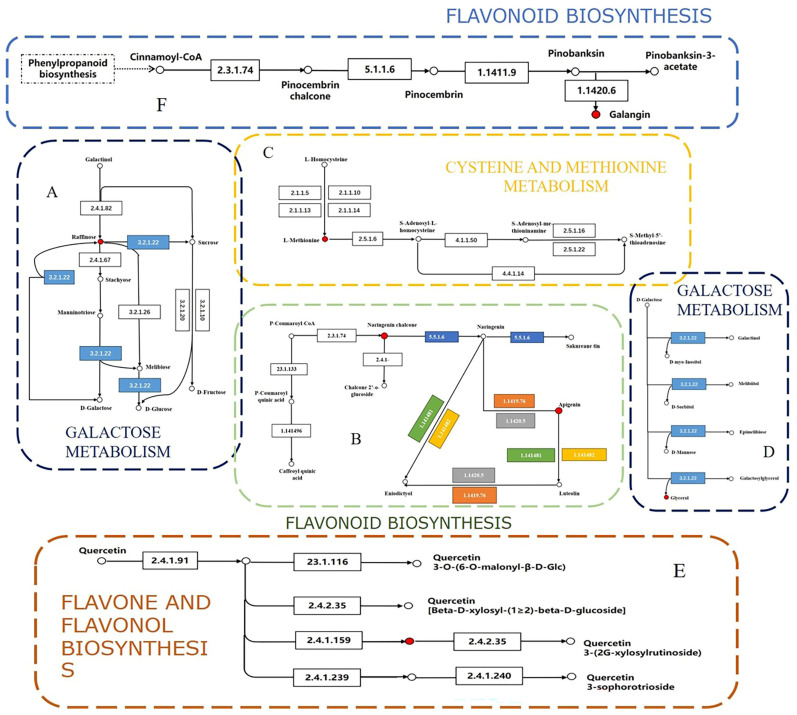
The key differential metabolites of DN and different species of *Dendrobium* were mapped to metabolic pathways, and the results of metabolic pathway analysis are shown as KEGG pathway results: (**A**,**D**) galactose metabolism; (**B**,**F**) flavonoid biosynthesis; (**C**) cysteine and methionine metabolism; (**E**) flavone and flavonol biosynthesis.

DN was compared to 170 key differential metabolites screened from other species of *Dendrobium*. A comparison with authoritative metabolite databases, such as KEGG and PubChem, revealed the regulatory pathways involved in these differential metabolites (Figure 5). The differential components primarily affect 6 pathways. Out of these, cysteine and methionine metabolism, flavonoid biosynthesis, galactose metabolism, and flavone and flavonol biosynthesis exhibit the greatest impact. This implies that the dissimilarities mostly occur within these four pathways (Figure 6).

## 3. Discussion

### 3.1. Differences in Compound Composition

Firstly, the total ion chromatograms of DT and DV, DC, and DO were comparable. However, there were significant differences found in the DN varieties of *Dendrobium*, particularly in the retention time bracket of 0–10 min. Notably, the peak intensity of DN varieties surpassed that of all other *Dendrobium* species, which indicated substantial variations in their chemical compositions. (2beta,3alpha,4beta,5beta,25R)-Spirostan-2,3,4-triyl triacetate and schisandrin B were detected in both the DN and DC species only and the signal intensity was notably stronger in DN than in DC. Polyphyllin VI was only detected in DN. In the case of *Dendrobium*, most of the literature considers the alkaloids and polysaccharides in DN to be the compound species that differentiate it from the others [41]. However, the differential compounds detected in this paper were dominated by terpenoid components. This suggests that if a further distinction is to be made between DN and other *Dendrobium* species based on pharmacological activity, the physiological activity of the terpenoid components in *Dendrobium* must be investigated.

### 3.2. Influence of Geography on the Chemical Composition of Different Dendrobium Species

*Dendrobium* is a genus of about 1000 species, widely distributed in tropical and subtropical areas of Asia and Oceania. Among them, there are 74 species and 2 varieties in China, which are produced in the provinces and districts south of the Qinling Mountains, especially in the south of Yunnan province [42]. In this article, seven of the most prevalent species were chosen for comparison, and it was established that the chemical composition of DN distinctly varies from that of the other six *Dendrobium* species. DN thrives in humid settings and is largely an epiphyte, excluding a few terrestrial species. The majority of plants are cultivated in southern China, resulting in a sizeable population. Although southern China falls within the subtropical region, the area is mainly characterized by a subtropical monsoon climate. This climate is marked by high temperatures and humid and rainy summers [43]. This may be the reason why the chemical composition of DN is different from that of other species of *Dendrobium*.

DN stems are tufted, slightly flattened at the top and slightly curved and ascending, 10–60 cm tall and up to 1.4 cm thick [44], whereas other *Dendrobiums* such as DO have erect cylindrical stems, 9–35 cm long, 2–4 mm thick, unbranched, with multiple nodes. In addition, the DN chloroplast genome has four typical regions—leukemia stem cell (LSC) (84,939 bp), spermatogonial stem cell (SSC) (13,310 bp), and two inverted repeats (IRs (26,272 bp each) [45]—which may also be a reason why the chemical composition of DN differs from that of other *Dendrobium* species. 

### 3.3. Comparison of DN Activity with Other Species of Dendrobium

There were 34 different components between DN and other species of *Dendrobium*, including 9 terpenoids, which accounted for the highest percentage; among the terpenoids, *Liriope platyphylla* (LPTS) total saponin improves memory, promotes body weight, and increases thymus and spleen indices in D-galactose-induced senescent mice. LPTS decreases Malondialdehyde (MDA) and lipofuscin levels, inhibits monoamineoxidase (MAO) activity, and increases Superoxide dismutase (SOD) activity and Glutathione reductase (GSH-PX) levels [46]. Tanshinone IIB is a major active constituent of the roots of Salvia miltiorrhiza (Danshen) widely used for the research of stroke and coronary heart disease in Asian countries, tanshinone IIB has a neuroprotective effect via inhibition of apoptosis [47]. Fimbriatone is a new compound, while physcion and rhein were first isolated from the genus *Dendrobium*. The others were found from this species for the first time. Fimbriatone showed potential inhibitory effects on BGC-1 [48]. The cysteine and methionine metabolism metabolic pathways are associated with coronary heart disease and antimicrobial and psychoneurological-related diseases such as Parkinson’s and Alzheimer’s [49,50,51]. Flavonoid biosynthesis is metabolically related to antioxidant and anti-atherosclerotic properties [52]. Galactose metabolism can inhibit uric acid synthesis, thus exerting a uric-acid-lowering uric acid-lowering effect [53].

## 4. Materials and Methods

### 4.1. Plant Materials, Instruments, and Reagents

Samples of seven *Dendrobium* varieties were collected in the same area during the same growing periods. Seven kinds of fresh *Dendrobium* strips were collected from Longling County, Yunnan Province, and were annotated as DD (SH-PZ1-202110), DC (SH-PZ2-202110), DN (SH-PZ3-202110), DF (SH-PZ4-202110), DT (SH-PZ5-202110), DO (SH-PZ6-202110), and DV (SH-PZ7-202110) by Professor Pengfei Tu of Peking University School of Pharmacy. The specimens of each variety of *Dendrobium* are currently preserved at the Institute of Food Science and Technology CAAS.

The following equipment was used in this study: an ultra-high performance liquid chromatograph-tandem mass spectrometer (Waters ACQUITY UPLC/Xevo G2 QTOF MS, Waters Technologies, Shanghai, China) with binary high-pressure pump, automatic sampler, and column temperature box; Progenesis QI 2.2 software (Waters company); SIMCA 14.1 data processing software; a Milli-Q ultrapure water meter (Millipore Company, Burlington, MA, USA); a high-speed multifunctional Chinese medicine pulverizer (Yongkang Boou Hardware Products Co., Ltd., Yongkang, China); a 5430R small centrifuge (Eppendorf company, Hamburg, Germany); a YQ-ZJ-107 electronic balance (Guangzhou Radio and Television Measurement and Testing Co., Ltd., Guangzhou, China); and an RE-52AA rotary evaporator (Shanghai Yarong Biochemical Instrument Factory, Shanghai, China).

The following reagents were used: acetonitrile and formic acid (batch number C12122679, chromatographic purity, Merck company, Germany); methanol (batch number 143135, chromatographic purity, Jiangsu Hanbang Technology Co., Ltd., Huaian, China). The water used in the experiments was ultrapure water prepared using the Milli-Q water purifier.

### 4.2. Sample Preparation

Samples for LC–MS analysis were prepared by taking a proper amount of the seven kinds of fresh *Dendrobium*, cutting the fresh *Dendrobium* strips into oblique slices with a thickness of about 3~5 mm, pre-freezing them at −18 °C for 10 h, drying them in a vacuum freeze-dryer, breaking them with a high-speed crusher, and sieving them with a 65-mesh sieve to obtain fresh *Dendrobium* strip powder. About 1.0 g of the seven kinds of fresh *Dendrobium* powder was accurately weighed and put into a 50 mL centrifuge tube. A volume of 30 mL of anhydrous methanol was added, then the tube was plugged tightly, shaken evenly, and weighed. Ultrasonic extraction (power 500 W, frequency 40 kHz) was performed for 30 min, followed by cooling, weighing, supplementing the weight loss with methanol, shaking, centrifuging (rotating speed 4000 r min^−1^) for 20 min, collecting the supernatant, concentrating to dryness, redissolving with methanol to 2 mg/mL, and filtering with a 0.22 μm microporous membrane to obtain DD, DC, DN, DF, DT, DO, and DV. Seven kinds of *Dendrobium* fresh strip solutions were made, with n samples of each in parallel (n ≥ 5).

### 4.3. UPLC-QTOF-MS/MS Spectrometric Conditions

Chromatographic conditions: chromatographic column: ACQUITY UPLC HSS T3 Column (2.1 mm × 100 mm, 1.8 μm); column temperature of 35 °C; mobile phase: 0.1% formic acid aqueous solution (A)–acetonitrile (B), gradient elution (0~15 min, 95~10% A; 15~18 min, 10~0% A; 18~23 min, 0% A; 23~23.01 min, 0~95% A; 23.01~28 min, 95% A); flow rate of 0.3 mL/min; sample injection volume of 5 μL.

Mass spectrometry conditions: ESI, power supply voltage, and spray voltage of 2.23 kV; sheath gas (nitrogen) flow rate of 28 arbs; auxiliary gas flow rate of 8 arbs; positive ion first-order full scanning mode; molecular weight range of *m*/*z* 50~1200. In addition, the mixed reference and mixed test solutions were analyzed by secondary mass spectrometry, which is beneficial to the identification of chromatographic peaks.

### 4.4. Data Processing, Statistical Analysis, and Identification of Metabolites

The UPLC-QTOF-MS raw data of the seven *Dendrobium* varieties were imported into Progenesis QI 2.3 software (Waters XBridge, Beijing, China). The intensity of each ion was normalized and filtered to the total ion count to generate a data matrix with an *m*/*z* value, Rt, and normalized peak area. We searched the chemical composition database, natural product database, and database of *Dendrobium* established in the early stage, explored the cracking rule according to the fragments from primary and secondary mass spectrometry, and identified the structure of the compounds by comparison with reference substances. The processed data were analyzed using SIMCA13.0 software (Umetrics, Umea, Sweden) for multivariate analysis. The relationships in the data matrix were derived using unsupervised PCA. Next, we used SIMCA13.0 software to perform OPLS-DA and investigate the differential metabolites between the two PF varieties [54]. To obtain this model, we set the scale to par and edited the model type as OPLS-DA. Selecting “Autofit” resulted in one prediction component and one orthogonal component. VIP analysis was conducted to evaluate the significance of metabolites and select those with the highest discrimination potential among the seven *Dendrobium* varieties (VIP ≥ 1) and a *p*-value of ≤0.05 (independent-samples *t*-test).

The metabolites that met these criteria were identified as differential metabolites. Based on their mass spectral data, their structures were annotated using the *Dendrobium* self-build library and relevant published literature, and they were confirmed against reference compounds or by their fragmentation patterns [19]. Heat maps of the differential metabolites across the seven *Dendrobium* varieties were generated using OriginPro 2017 software. The peak areas were normalized, processed, and imported into OriginPro 2017 software, with color bands adjusted to produce a clear visual representation.

## 5. Conclusions

*Dendrobium*, a frequently used Chinese medicine, displays significant differences in its application and the amount used, depending on the species of *Dendrobium* plant in question. In this study, 242 compounds from numerous *Dendrobium* varieties were analyzed using a metabolomics method based on UPLC-QTOF-MS/MS, in conjunction with a literature review. Subsequently, PCA and OPLS-DA were conducted to identify differentially labeled metabolites amongst the aforementioned varieties, indicating variance in the chemical composition of the *Dendrobium* cultivars.

In this paper, UPLC-MS, multivariate statistical analyses, and other means were used to determine the differences between DN and other species of *Dendrobium*. The analysis revealed that the differing metabolites present in DN and other species of *Dendrobium* were predominantly terpenoid and flavonoid components. According to recent research, terpenes, and flavonoids have a good effect on cardiovascular diseases, which coincides with their traditional functions for delaying aging, promoting blood circulation, and removing blood stasis. In all, 170 different metabolites were screened, and this analysis revealed six relevant pathways, including cysteine and methionine metabolism, flavonoid biosynthesis, and galactose metabolism. Consequently, it can be inferred that the dissimilarities between DN and other *Dendrobiums* are primarily associated with these three pathways of metabolite synthesis. 

## Figures and Tables

**Table 1:** Differential composition of *D. nobile* vs. other *Dendrobium* species.

Component	t_R_ (min)	*m*/*z*	Mass Error (ppm)	Type
Naringenin chalcone	5.15	273.0766	3.2783	flavonoid
Dehydrocostus lactone	5.54	231.1386	2.5888	sesquiterpene lactone
Salsolinol	5.98	180.1028	4.8397	phenolic acid
Polyphyllin VI	6.58	267.1597	1.2508	saponins
4′-Dihydroabscisic acid	6.58	267.1597	2.1435	phenolic acid
(-)-dendroresinol	6.61	250.1802	2.0007	phenolic acid
Trichothecene isoflavone-7-*O-β*-D-glucoside	6.87	167.0707	3.7294	glucoside
Tanshinone I	7.19	277.0861	0.9370	abietane diterpenoid
Arglabin	8.55	310.2018	2.6096	sesquiterpene lactone
α-Cyperone	8.71	218.3357	3.4204	sesquiterpenoids
Allocryptopine	8.83	370.1665	4.2421	alkaloid
Isopetasoside	8.96	201.1643	6.4600	terpene glycoside
Dimethoxyphenanthrene diglucose	9.47	219.1748	4.3400	phenanthrene
(+)-Nootkatone	9.47	219.1748	2.1705	sesquiterpenoid
Atractylenolide III	10.40	249.149	1.7823	naphthofuran
4-(3-hydroxy-4-methoxyphenethyl)-2,6-dimethoxylphenol	11.08	251.1644	7.6273	phenolic acid
(2beta,3alpha,4beta,5beta,25R)-Spirostan-2,3,4-triyl triacetate	11.08	189.1638	0.9266	triacetate
Flavokawain B	12.56	285.1131	3.2482	flavonoid
Rheidin B	12.62	278.176	2.7592	glucoside
Fimbriatone	12.64	283.0615	6.8948	terpenoids
Chrysoeriol	13.20	301.0726	8.1728	phenolic acid
Tanshinone IIB	13.26	297.1495	3.2990	terpenoid
Baohuoside I	16.20	515.1937	4.8857	terpenoids
Levistilide A	16.34	381.2067	1.7695	coumarins
Berberrubine	17.61	322.1085	3.5565	alkaloid
Neoruscogenin	17.67	429.3001	0.4801	sapogenin
Sarsasapogenin	17.96	869.4946	4.0822	sapogenin
Licochalcone C	18.06	339.1603	3.4968	flavonoid
Heteroclitin D	18.17	483.202	1.4311	lignin
7-hydroxy-9,10-dihydro-1,4-phenanthrenedione	19.00	389.1245	2.0699	phenanthrene
Schisandrin B	19.18	401.1966	1.9350	phenanthrenes
Retrochalcone	21.79	271.0972	2.7592	flavonoid
Volemolide	27.81	347.2597	4.5732	terpene lactone
Homodolichosterone	31.64	477.3594	4.0064	steroid

All compounds collection adducts are [M + H]^+^.

## Data Availability

Data are contained within the article or Supplementary Materials.

## Supplementary Materials

The following supporting information can be downloaded at: https://www.mdpi.com/article/10.3390/ijms242417148/s1.

## Abbreviations

Abbreviation	Name
DD	*Dendrobium denneanum* Kerr.
DC	*D. chrysotoxum* Lindl.
DN	*D. nobile Lindl.*
DF	*D. fimbriatum* Hook.
DT	*D. thyrsiflorum* Rchb. f.
DO	*D. officinale* Kimura. et Migo.
DV	*D. devonianum* Paxton.
ESI	Electrospray ion source
BPI	Base peak ion
PCA	Principal component analysis
OPLS-DA	Orthogonal partial least-squares discrimination analysis
CE	Collision energy
VIP	Variable importance in projection
UPLC-QTOF-MS/MS	Ultra-performance liquid chromatography–electrospray ionization–tandem mass spectrometry
KEGG	Kyoto Encyclopedia of Genes and Genomes
MDA	Malondialdehyde
SOD	Superoxide dismutase
GSH-PX	Glutathione reductase
BGC-1	Bovine granulosa cell line
