# Supplementary material for: Comparison of the Metabolomics of Different Dendrobium Species by UPLC-QTOF-MS
Source: Int J Mol Sci. 2023 Dec 5;24(24):17148. doi: 10.3390/ijms242417148 (PMC10742841; doi:10.3390/ijms242417148)
Supplement: Supplementary file 1 [file ijms-24-17148-s001.zip › Figures S1-S3.pdf]

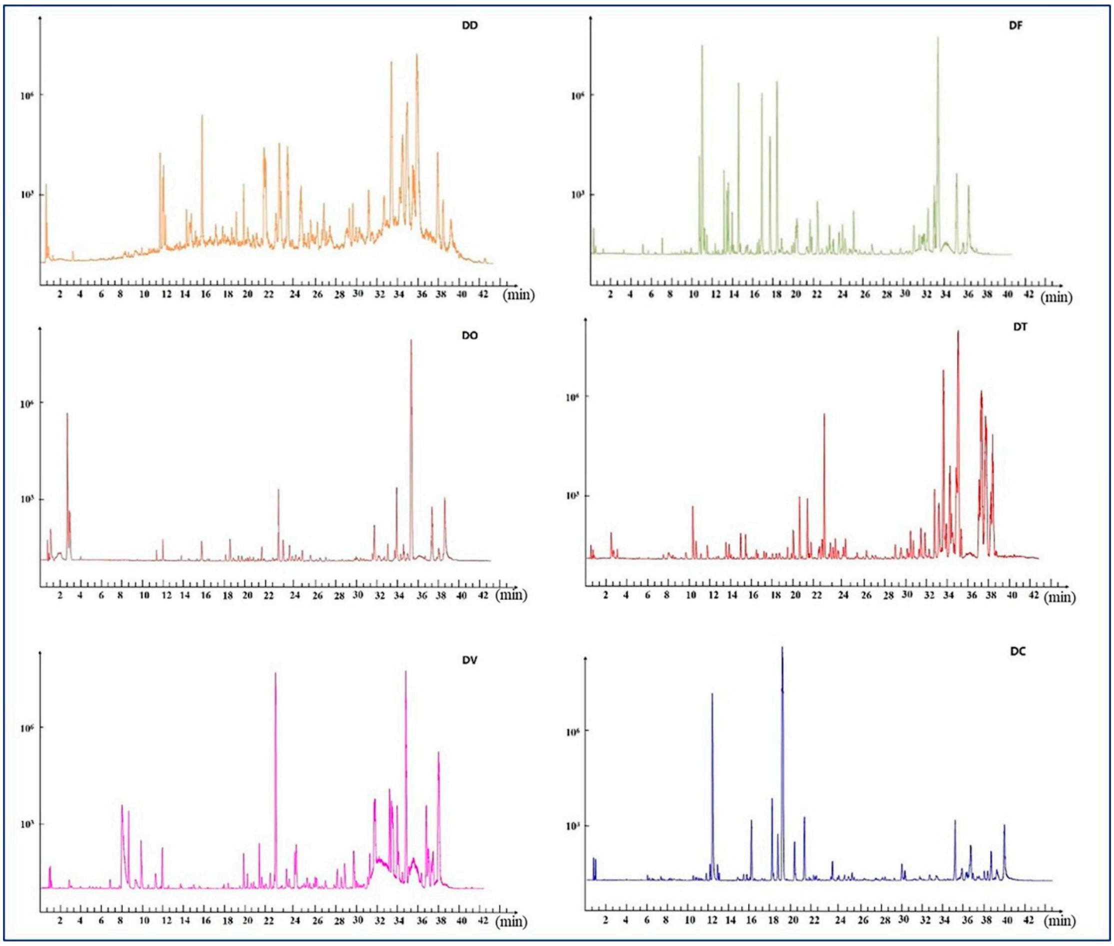

Figure S1. Total ion current (TIC) chromatograms of 6 *Dendrobium* species in positive ion.

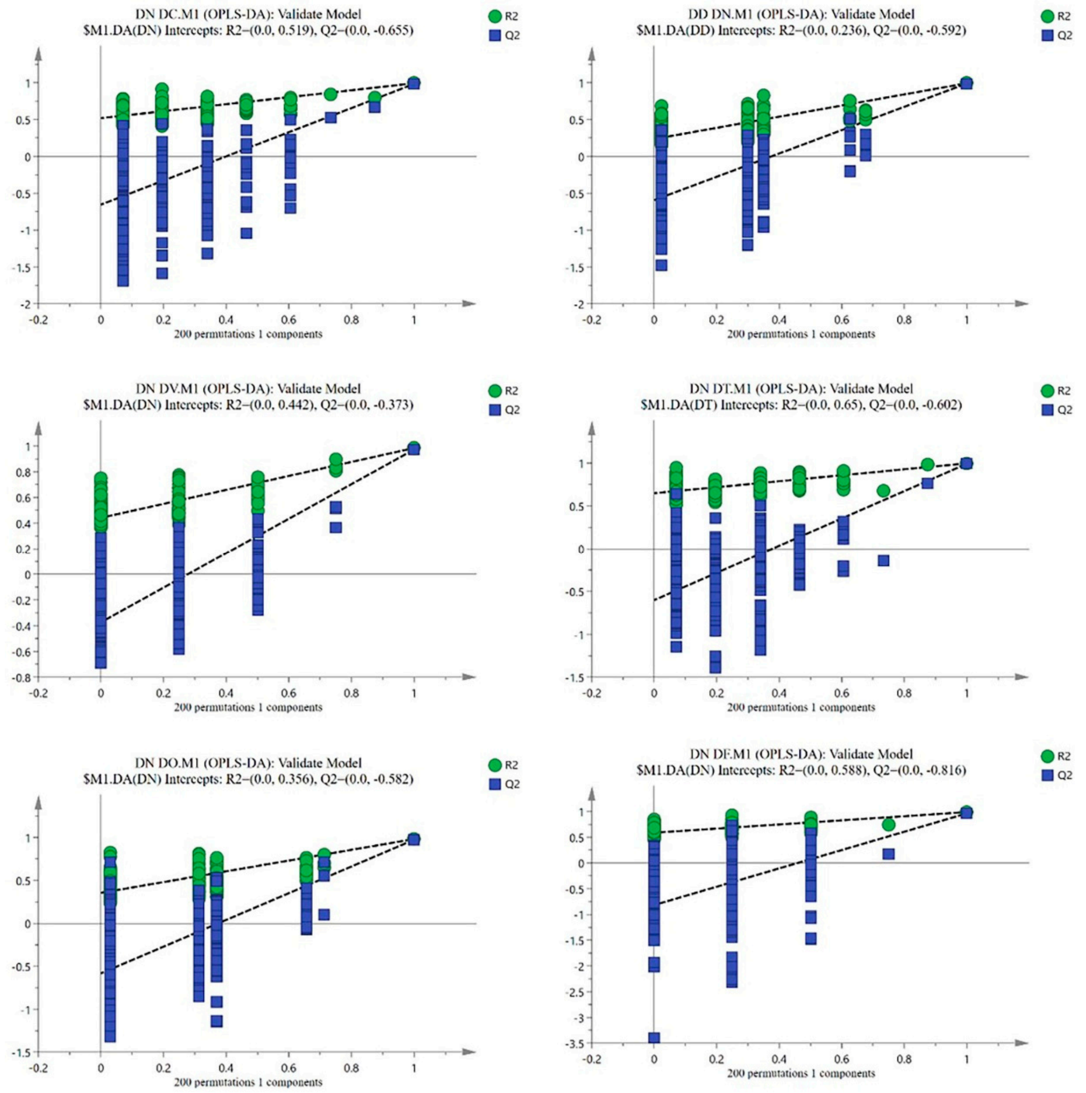

Figure S2. Comparison of *D. nobile* with other species of *Dendrobium* OPLS-DA Validation Chart.

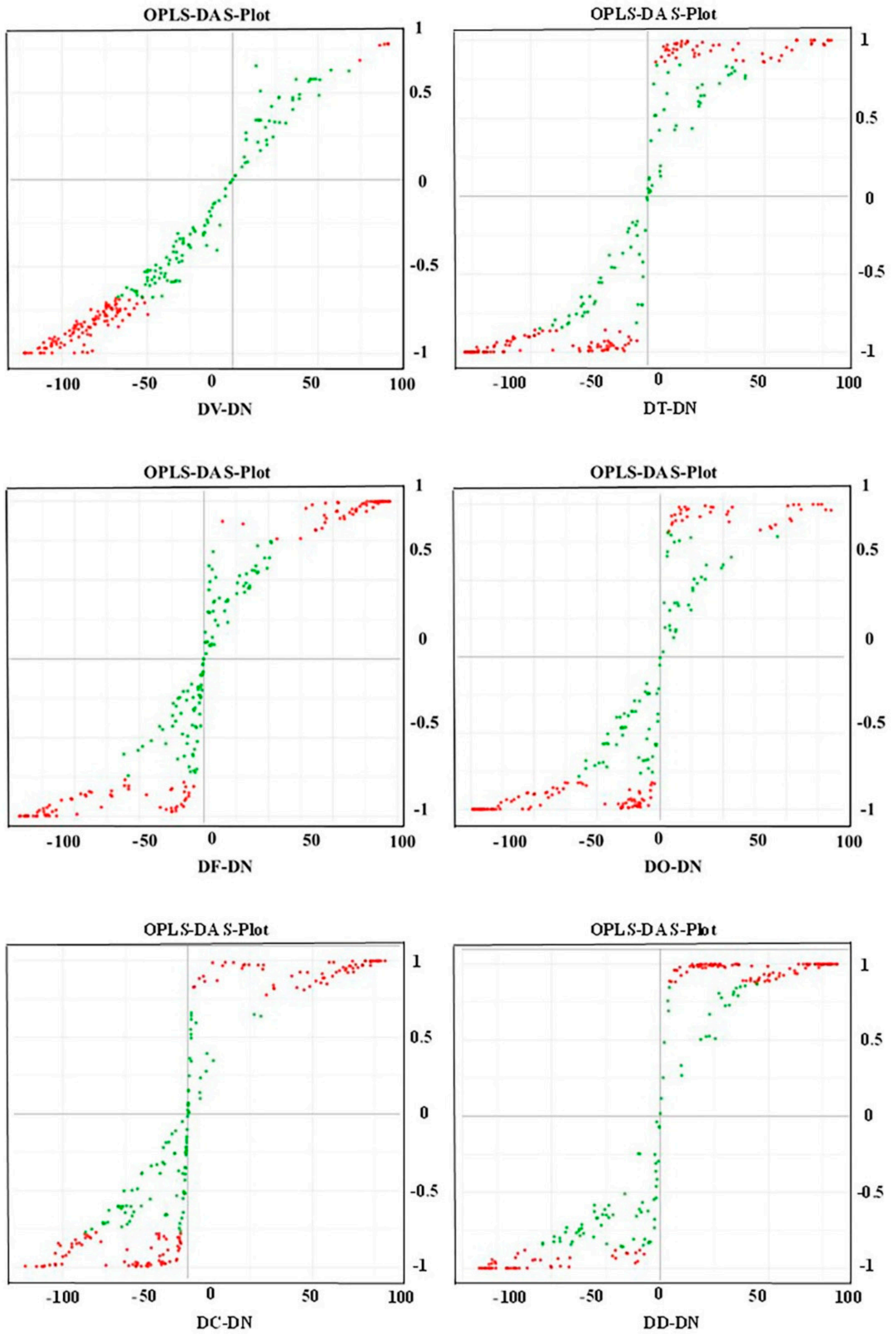

Figure S3. Comparison of *D. nobile* with other species of *Dendrobium* OPLS-DA S-plot.
